# ModBind, a Rapid Simulation-Based Predictor of Ligand Binding and Off-Rates

**DOI:** 10.1021/acs.jcim.4c01805

**Published:** 2024-12-16

**Authors:** William Sinko, Blake Mertz, Takafumi Shimizu, Taisuke Takahashi, Yoh Terada, S. Roy Kimura

**Affiliations:** † Alivexis Inc., 1 Broadway, 14th Floor, Cambridge, Massachusetts 02142, United States; ‡ Alivexis Inc., Daiichi Hibiya Building 7F, Shimbashi 1-18-21, Minato-ku, Tokyo 105-0004, Japan

## Abstract

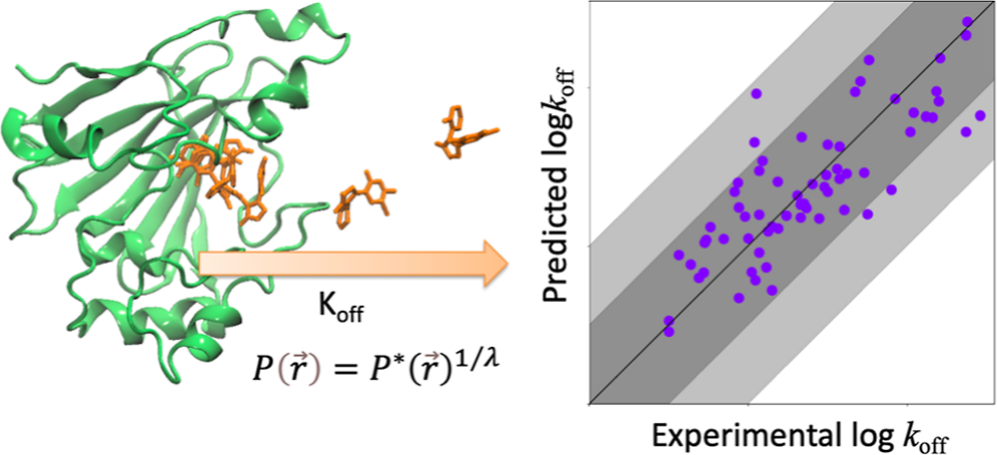

In rational drug discovery, both free energy of binding and the binding half-life (*k*_off_) are important factors in determining the efficacy of drugs. Numerous computational methods have been developed to predict these important properties, many of which rely on molecular dynamics (MD) simulations. While binding free-energy methods (thermodynamic equilibrium predictions) have been well validated and have demonstrated the ability to drive daily synthesis decisions in a commercial drug discovery setting, the prediction of *k*_off_ (kinetics predictions) has had limited validation, and predictive methods have largely not been deployed in drug discovery settings. We developed ModBind, a novel method for MD simulation-based *k*_off_ predictions. ModBind demonstrated similar accuracy to current state-of-the-art free-energy prediction methods. Additionally, ModBind performs ∼100 times faster than most available MD simulation-based free-energy or *k*_off_ methods, allowing for widespread use by the molecular modeling community. While most free-energy methods rely on relative free-energy changes and are primarily useful for optimization of a congeneric series, our method requires no structural similarity between ligands, making ModBind an absolute predictor of *k*_off_. ModBind is thus a tool that can be used in virtual screening of diverse ligands, making it distinct from relative free-energy methods. We also discuss conditions that enable approximate prediction of ligand efficacy using ModBind and the limitations of this approach.

## Introduction

Binding half-life drives pharmacodynamic effects in vivo after drug concentration drops, and longer residence times can improve both selectivity and in vivo efficacy of drugs.^1,2^ Although binding affinity and free energy of binding are important properties that are often the main considerations in lead discovery and optimization, any drug discovery program that focuses solely on the optimization of free energy of binding is inadequate, as pharmacodynamic effects may greatly affect drug efficacy due to changes in drug concentration over time. In the area of free-energy prediction, significant academic and commercial works have demonstrated that with reasonable structural information, current state-of-the-art methods can predict the relative free-energy differences between a congeneric series of ligands with good accuracy using validation sets of hundreds of compounds that bind targets across a broad range of target classes. The accuracy is generally less than 1 order of magnitude and often close to half an order of magnitude for the best free-energy methods.^3−7^ This provides excellent predictivity to help drive synthetic decisions in the hit-to-lead and lead optimization phases of drug discovery.^8,9^ In contrast to binding affinity, the prediction of kinetic on and off rates (*k*_on_ and *k*_off_) has received considerably less attention from the computational chemistry field.

Although several methods to predict *k*_off_ have been published, the validation of these approaches has been limited, and few demonstrations of commercial drug discovery impact have been published.^10^ The majority of these methods employ some type of enhanced sampling approach to drive the dissociation of the ligand. Tau-random accelerated molecular dynamics (τ-RAMD) applies a random force to the ligand, facilitating dissociation from the protein-binding pocket.^11,12^ Steered MD applies a constant force to the ligand to drive displacement; the force must be defined along a vector.^13−15^ Targeted MD drives a system from an initial state to a final state (i.e., the target) by applying a predefined force to the coordinates of the initial system and has been used to map ligand dissociation pathways.^16,17^ Ligand Gaussian-accelerated MD (LiGaMD) applies a boost potential to specific portions of the system (ligand, dihedral bonds, and overall potential energy) to drive binding and unbinding of the ligand from the protein target, allowing for determination of both *k*_on_ and *k*_off_.^18,19^ Metadynamics has been used to model ligand dissociation by applying a Gaussian boost to the energy surface along a defined collective variable that corresponds to the unbinding pathway.^20,21^ Finally, milestoning has been used to predict ligand-binding kinetics by dividing the phase space of ligand–protein complex formation into cells separated by milestones and simulating a series of transitions to predict the overall kinetics.^22^ Although each of these approaches can be robust with respect to modeling ligand dissociation, they all suffer to some degree from a combination of being computationally expensive, requiring prior knowledge of the protein system and binding or unbinding pathway and involving complex computational setups and workflows. In addition, public data sets of *k*_on_ and *k*_off_ are much less available than binding affinities, and in practice, the binding half-life of compounds is measured less routinely than binding or inhibition constants (which can be directly related to relative free energy through the Cheng–Prusoff equation). This might further prevent the development and validation of the *k*_off_ prediction methods.

Here, we present a new method for *k*_off_ prediction, ModBind, supported by validation with a robust data set. The approach employs high-temperature MD simulations to drive ligand dissociation that would otherwise occur on time scales (ms to hr) far beyond those accessible with equilibrium MD. Additionally, the prediction workflow is more efficient than current free-energy-based protocols, making ModBind suitable for high throughput hit finding while being accurate enough for hit-to-lead and lead optimization. More importantly, unlike most implementations of free-energy perturbation, ModBind is an absolute *k*_off_ predictor that does not require a reference compound with a known *k*_off_. The setup requires only three-dimensional coordinates of the biological target and a reasonable model for the ligand binding mode, both of which are basic prerequisites for any structure-based drug discovery campaign.

## Theory

Previously, one of the authors proposed a simple method for accelerated sampling and reweighting of the conformational landscape for a given molecular simulation.^23^ This method has advantages in that the reweighting scheme is not dependent on the fluctuating energy of the system but rather on the distribution of conformational states of the system, which is often more interpretable and scalable to larger systems. We build on this theory in the development of ModBind. Mollica et al. described an adaptation to this population-based reweighting scheme that provided the theoretical framework to calculate *k*_off_.^24,25^ We further developed this population-based reweighting theoretical framework and significantly improved the speed and accuracy of the protocol to create ModBind.

While running MD simulations on a scaled potential energy surface is known to accelerate the rate and scope of sampling conformational space,^26,27^ this practice also alters the free-energy landscape. Thus, the correlation between the real distribution of sampled conformations and the partition function is altered. The population at equilibrium for a configuration (or microstate), (), depends only on the potential energy (*V*) at that point
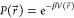
1

Similarly, in the case of a simulation conducted on a scaled temperature potential, P*()
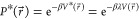
2where β = (*k*_B_*T*)^−1^ with *k*_B_ as the Boltzmann constant and *T* as the temperature and λ is a user-defined scaling factor. When λ is applied to a given system, an altered ratio of the populations of microstates is produced. From eqs 1 and 2, we see that there is a simple relation between the population of a scaled MD run, P*(), and the population, *P*(), corresponding to a Boltzmann distribution along the original potential
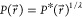
3

By running simulations with a scaled potential, sampling can be significantly enhanced. The relationship in eq 3 can then be applied to recover the Boltzmann distribution and energies of any microstate () including the (presumed) bioactive conformation.

Mollica et al. expanded the theory in eq 3 to predict dissociation rates of protein–ligand systems, showing that *k*_off_ of ligands could be predicted relative to other ligands^24^
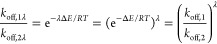
4

We used eq 4 but with high temperatures to accelerate ligand-unbinding events. In the original publication introducing this scaled potential, it was noted that temperature could be used as the scaling factor and that the same reweighting scheme could be used to recover the correct distribution at the unscaled temperature.^23^

## Methods

### System Setup

All protein structures were obtained from the Protein Data Bank (http://www.rcsb.org) unless otherwise specified. A list of the protein structures used can be found in the Supporting Information. Protein structures were prepared using the Schrodinger protein preparation workflow^28^ to model in missing residues, determine tautomeric and p*K*_a_ states, and perform a restrained minimization to optimize the structure before docking. If a known binding mode existed for a ligand to the protein target, 3D alignment or restrained docking was used. For targets without a known related ligand-binding pose, docking was performed with ligands restricted to experimentally validated binding sites. Glide^29,30^ or AutoDock Vina^31^ was used to generate all ligand–receptor poses unless otherwise noted. Ligand force-field parameters were generated following the protocol implemented in OpenMM,^32,33^ which uses the openmmforcefields package^34^ to develop parameters consistent with the general Amber force field 2.1.^35^

### Simulation Parameters

Temperatures in this study ranged from 600 to 1000 K and were maintained with the Langevin thermostat.^36^ Individual simulation times depended on the rate of ligand unbinding, typically 1–5 ns or less. Temperatures and simulation times were optimized by running simulations on known inhibitor(s) and calculating the corresponding *k*_off_ rates. Simulations were conducted in the *NVT* ensemble with a 2 fs time step using OpenMM7.^26^ Backbone atoms of the protein were restrained (default value of σ = 3.0 Å but may need to be adjusted depending on the system) except for residues within 6 Å of the protein binding site. The use of restraints is applied to prevent protein unfolding prior to unbinding, which likely would result in a poorer prediction accuracy. Mollica et al.^24^ and ModBind have used restraints in unbinding simulations without seeing deleterious effects on either the prediction accuracy or the ability of a ligand to unbind across many targets. While we have not encountered issues with the restraints, there may be cases where restraining the protein could prevent the ligand from fully unbinding if the pathway is blocked by restrained protein residues. In this case, restraints may need to be adjusted or not used. Up to 32 replicate trajectories were run to generate sufficient statistics for the reliable calculation of *k*_off_ rates.

### Analysis

Pytraj,^37^ a Python package binding to the cpptraj program,^38^ was used for postprocessing simulations. Trajectory frames were aligned to the first frame (protein-to-protein) for each respective trajectory. Time-dependent root-mean-square deviation (rmsd) was then calculated for each respective ligand, with a 5.0 Å cutoff to classify ligands in the bound/unbound state. The raw unbinding rate was obtained by calculating the median simulation time for unbinding for all trajectories of a respective ligand, producing a *k*_off_ value at high temperature. Reweighting is carried out using eq 4 with room temperature (*T* = 300 K), producing the relative dissociation rate (*k*_off_) per ligand.

## Results and Discussion

### Initial Validation of the ModBind Approach: FAK, HSP90, and p38 MAP Kinase

**Figure 1:**
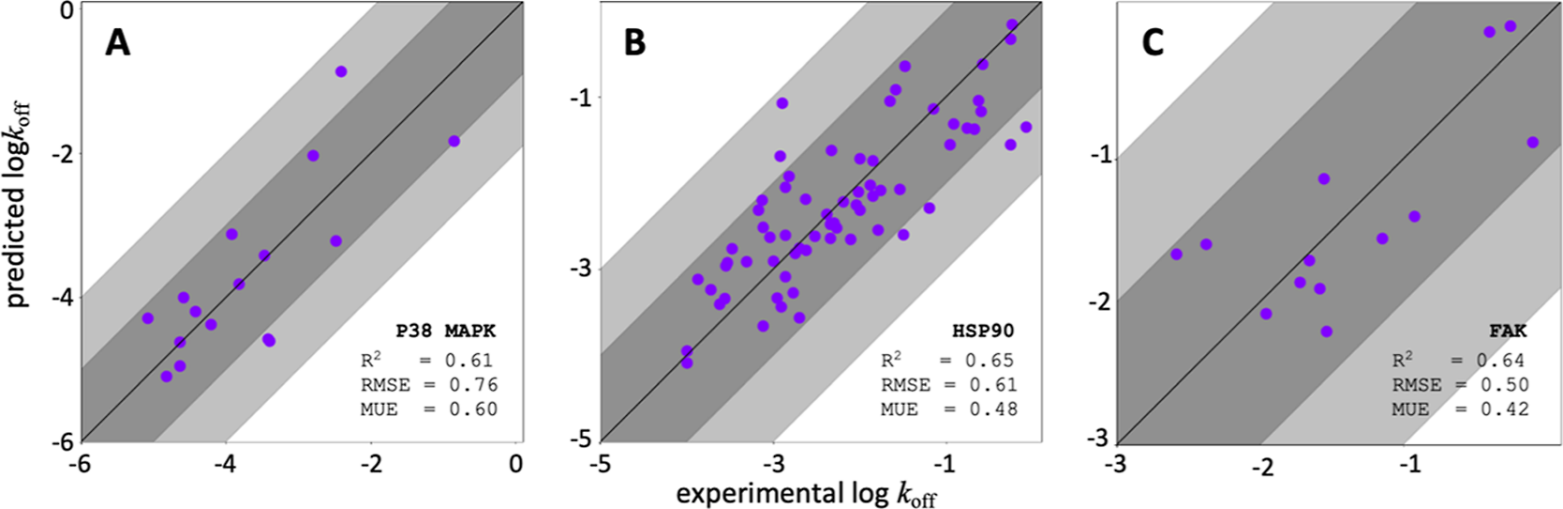
Experimental log(*k*_off_) versus ModBind predictions for targets with a wide range of dissociation rates. (A) p38 MAPK, (B) HSP90, and (C) FAK. Log(*k*_off_) values predicted by ModBind were normalized to allow for direct comparison to experiment. Dark-gray shading indicates less than 1 log order error, and light-gray shading indicates less than 2 log order error.

Validation of our approach required curation of an experimental data set measuring *k*_off_ values for receptor–ligand complexes. The first set of compounds selected was from a study conducted by Boehringer Ingelheim on p38 MAP kinase (Supporting Information Figure 1).^39^ Experimental *k*_off_ rates for the p38 MAPK study had a range of 5 orders of magnitude (10^–1^ to 10^–6^ s^–1^), which provides a good range to demonstrate that ModBind can distinguish between ligands with fast and slow off-rates. Simulations with *T* = 1000 K and *t* = 2 ns were required to observe the unbinding of all ligands. We observed good agreement with experiment, with 13 of 16 data points less than one standard deviation from experimental values and the other three data points within two standard deviations of expected values (Figure 1A).

A second target, heat shock protein 90 (HSP90), was included to test a system with multiple distinct structures and highly flexible loops.^11,12^ This was a larger data set (70 ligands) (Supporting Information Figure 2) with a similar range of *k*_off_ rates (10^–0^ to 10^–4^ s^–1^). ModBind settings of *T* = 900 K and *t* = 1 ns were sufficient to observe unbinding of all of the ligands. Similar agreement existed between the simulation and experiment for the HSP90 data (Figure 1B). Additionally, we included a third data set for focal adhesion kinase (FAK) which contained 14 ligands (Supporting Information Figure 3).^13,40^ Again, the predicted results were well correlated between predicted *k*_off_ and experimental values (Figure 1C). In total, for the 96 ligands across three targets, ModBind achieves highly competitive performance relative to other industry-standard simulation-based predictions of ligand efficacy, with high correlation observed between simulation and experiment (*R*^2^ = 0.61–0.65) with an RMSE = 0.50–0.76 log(*k*_off_) units and an MUE = 0.42–0.60 log(*k*_off_) units.

**Figure 2:**
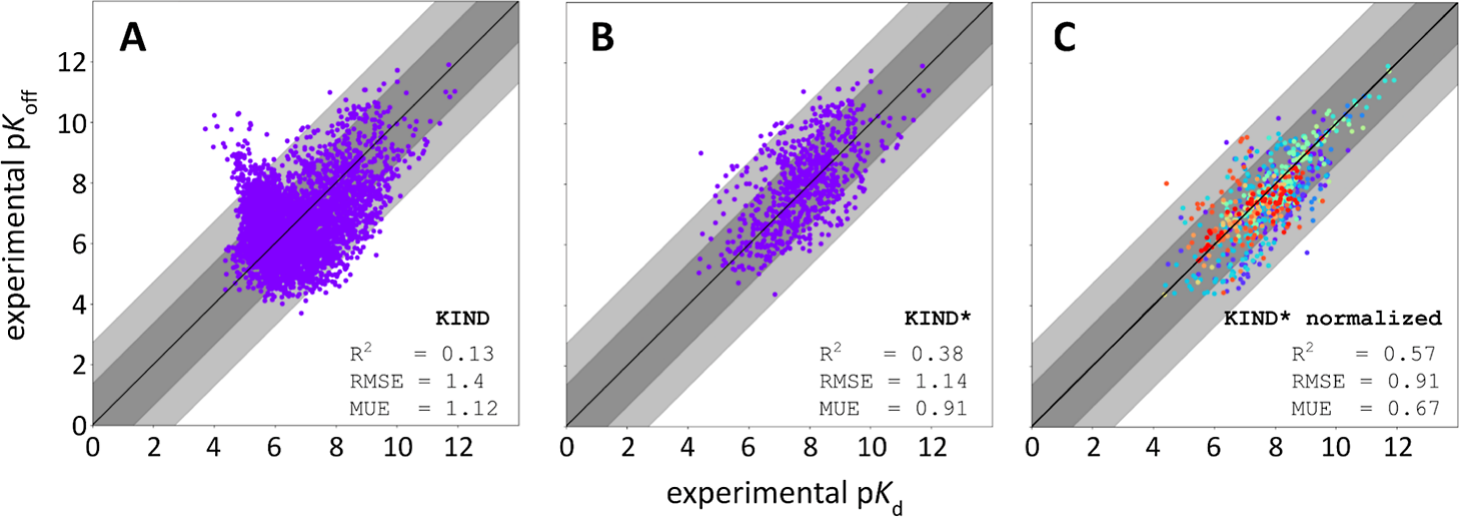
*k*_off_ shows good correlation with *K*_d_ for for filtered KIND dataset. (A) Relationship between log(*K*_d_) and log(*k*_off_) for the entire KIND data set including diverse (types 1 and 2) kinase inhibitors as well as data for InhA, which is optimized for correlation of *K*_d_ with Kon B. (B) Relationship between log(*K*_d_) and log(*k*_off_) for curated KIND data set removing data sets involving type 1 and 2 kinase inhibitors as well as data for InhA (KIND*). (C) Relationship between log(*K*_d_) and log(Kon) for the curated KIND data set removing data sets involving type 1 and 2 kinase inhibitors as well as data for InhA (KIND*); however, the data has been normalized by the target. Dark-gray shading indicates less than 1 log order error, and light-gray shading indicates less than 2 log order error. All data is adapted from Schuetz et al.^41^

Under certain conditions, ModBind scores are correlated with and may be applied toward the prediction of experimental ligand efficacy. Although we have demonstrated that ModBind can reproduce *k*_off_ values to a good accuracy, most small-molecule experimental assays performed in drug discovery research focus on quantifying a measure of ligand efficacy such as binding affinity, either by the equilibrium dissociation constant (*K*_d_) or by IC_50_/EC_50_ (drug concentration producing half-maximal inhibition or effect). *K*_d_ is related to the free energy of binding (Δ*G*) by
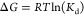
5where *R* is the gas constant and *T* is the temperature. The utility of ModBind can be extended by developing the ability to extrapolate trends in *k*_off_ to the binding affinity data. *K*_d_ is related to kinetic on and off rates by the following equation
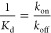
6i.e., *k*_on_ is inversely correlated with *K*_d_ and *k*_off_ is directly correlated with *K*_d_. Although this is true in theory, empirically, the strength of these correlative relationships can vary and is system dependent.^41^ The most relevant previous study on the relationship between *K*_d_, *k*_on_, and *k*_off_ focused on type-1 and type-2 kinase inhibitors (Table 1).^42^ The overall conclusion from this original work was that *k*_off_ was not closely correlated to *K*_d_ (*R*^2^ value equal to 0.13; Figure 2A).

**Table 1:** Ligand Statistics and Results for Schuetz et al. and This Study

	Schuetz et al.^41^	this work
no. of ligands	3812	762
no. of targets	78	29
no. of distinct Murcko scaffolds	490	285
*k*_off_ vs *k*_d_ correlation	*R*^2^ = 0.13	*R*^2^ = 0.57

Our additional analysis on this data set revealed that *k*_off_ and *K*_d_ are well correlated with *K*_d_ under certain reasonable assumptions for drug discovery programs; i.e., removal of (1) type-1 and type-2 kinase inhibitors for the same target^41^ and (2) inhibitors that focus on transition-state manipulation^43^ results in an improved *k*_off_ (Figure 2B). Furthermore, if we normalize the range of *k*_off_ and *K*_d_ to be equal to compensate for individual target differences, the correlation is significantly improved (Figure 2C), indicating that typical medicinal chemistry project work within a series of compounds is likely to have *k*_off_ and *K*_d_ well correlated. The normalization of the range is not possible for singleton data, so 38 targets with a single data point needed to be removed.

The improved correlation between *K*_d_ and *k*_off_ is likely the result of focusing our analysis on a series of compounds that have similar binding modes and excluding cases where a given protein target would likely adopt significant conformational changes across a series of ligands, e.g., ligands that bind to the active or inactive state of a kinase. Although, strictly speaking, a correlation between *k*_off_ and *K*_d_ is expected only if *k*_on_ is constant across the ligands examined; here, we empirically observe such a correlation across many compounds and a variety of target classes involving likely similar binding modes and protein conformations within each series. Others have shown that the dissociation free energy as calculated by metadynamics is also closely correlated with the binding free energy for congeneric series of small molecules.^20^ It is helpful to note that the KIND* data set (Figure 2B,C) is composed of both congeneric and noncongeneric series for each target, indicating that strong correlation is common even for noncongeneric series assuming the two conditions we set forth in the filtering of the KIND data set.

Based on our observation that *k*_off_ is empirically correlated to *K*_d_ for a large and diverse set of ligands, we decided to test ModBind’s ability to directly predict *K*_d_ and compare its accuracy against the most widely used approach in industry for binding affinity prediction, namely, relative free-energy calculations. The study by Wang et al.^3^ has been widely used for testing and validation of relative free-energy-based approaches in both industry and academia. We chose 6 of the 8 targets from the study of Wang et al.^3^: CDK2, thrombin, p38 MAPK, JNK1, TYK2, and MCL1 (Supporting Information Figures 4–9). We excluded two targets (BACE and PTP1B) because they are known to be challenging to absolute efficacy methods due to loop rearrangement and high protein reorganizational energies (PTP1B) and titration of key residues upon binding (BACE).^44^

**Figure 3:**
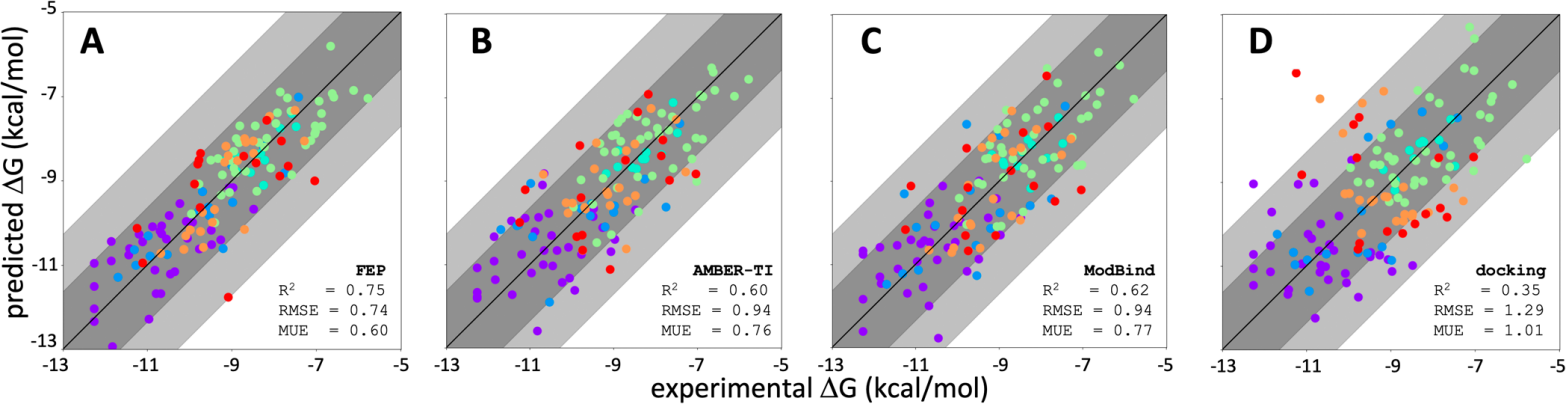
ModBind prediction of small-molecule binding energies is competitive with more expensive free-energy approaches. Experimental versus computationally predicted free energies of binding for TYK2 (blue), thrombin (cyan), MCL1 (lime), JNK1 (orange), CDK2 (red), and p38 kinase (purple), experimental data for the six protein targets shown were taken from Wang et al., JACS 2015.^3^ (A) Obtained using the FEP implementation in Desmond. (B) Experimental versus computationally predicted free energies of binding obtained using the TI implementation in AMBER18.^5^ (C) Experimental versus computationally predicted free energies of binding obtained using ModBind. (D) Experimental versus computationally predicted free energies of binding obtained using docking. Dark-gray shading indicates less than 1 log order error, and light-gray shading indicates less than 2 log order error.

Temperature scans were performed to identify the optimal temperature to produce a dynamic range of *k*_off_ values within a time frame of ≤4 ns per replica. As a benchmark, we compared the performance of ModBind against the results of the FEP method in Wang et al.^3^ and a comparable academic study using the thermodynamic integration (TI) implementation in AMBER18.^5^ The work with AMBER-TI also used the GAFF force field, as did our ModBind work. After normalizing scores from the FEP, AMBER TI, ModBind, and docking runs to free energies (i.e., setting the equivalent free-energy ranges for each protein target), we demonstrate that ModBind exhibits comparable accuracy with AMBER TI (Figure 3). Normalization requires prior knowledge of the experimental free energies of some or all ligands and is required to understand absolute potencies for ModBind or relative free-energy calculations but does not affect rank ordering or prioritization of ligands. We next compared to the original FEP work published by Wang et al.;^3^ the correlation and error metrics of FEP were improved compared to either ModBind or AMBER-TI. AMBER-TI and FEP are relative prediction methods, while ModBind is an absolute predictor which is generally more challenging to achieve high accuracy. We speculate that the higher correlation and lower errors observed for FEP for this validation set compared to both ModBind and AMBER-TI may be at least in part due to force-field differences (OPLS for FEP, GAFF for AMBER-TI and ModBind). Future work on ModBind will investigate the change in accuracy when using other force fields, and we expect the method to benefit from ongoing work to improve force fields. Additionally, we determined the correlation metrics using static structure docking. As expected, all of the free-energy methods and ModBind showed significantly better performance than docking.

ModBind can achieve a high degree of enrichment in small-molecule virtual screening (VS). One of the most promising applications of ModBind in drug discovery is structure-based VS. Although the advent of ultralarge commercially accessible compound libraries on the billion-compound scale^45−47^ makes computational screening an attractive option for hit-finding, the traditionally low experimental hit rates from structure-based and ligand-based screens make it difficult to reduce the virtual hit lists down to a manageable number of compounds.

**Figure 4:**
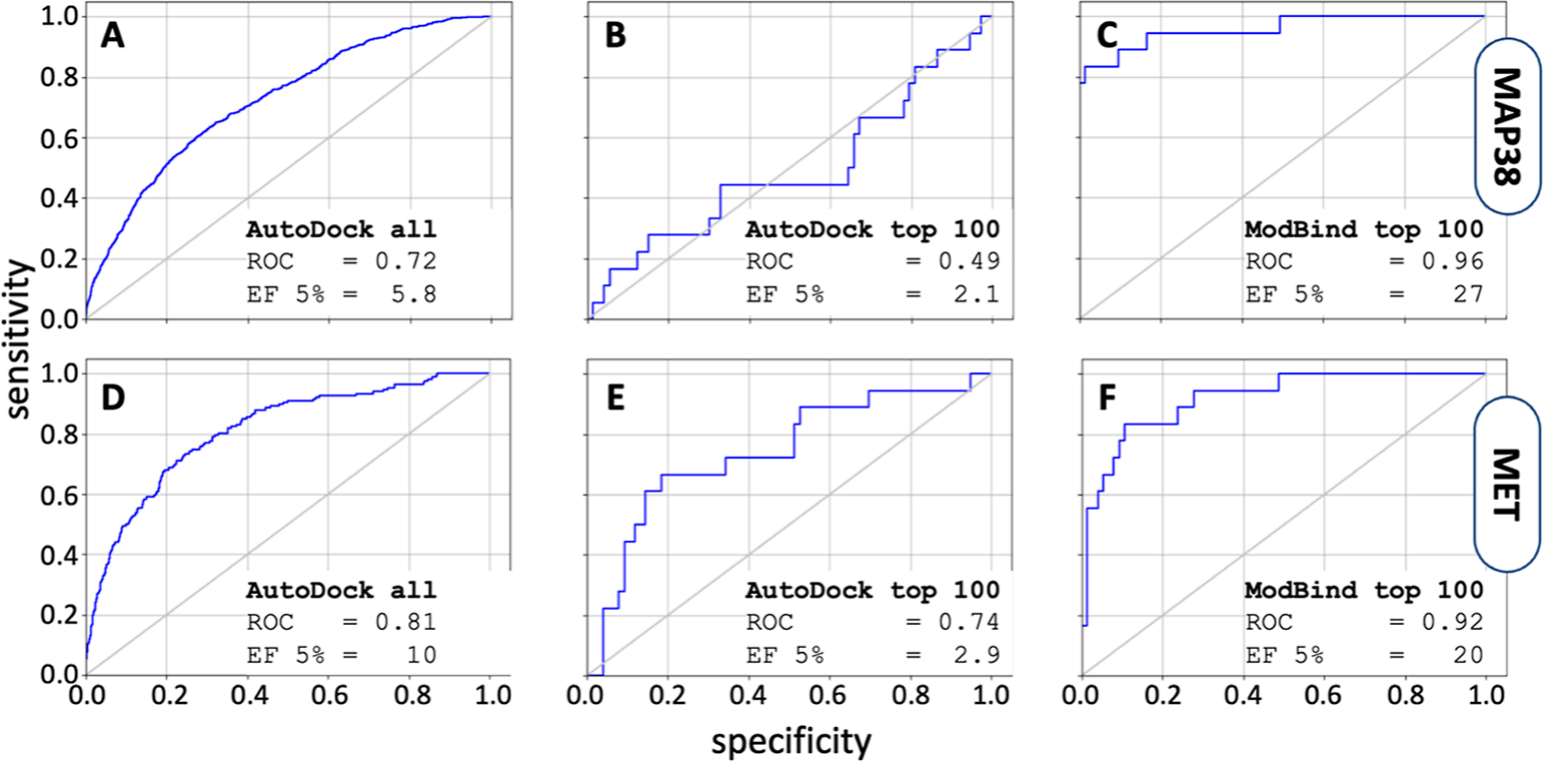
ModBind significantly improves enrichment to correctly identify active molecules from a VS campaign. (A) Enrichment curve for docking of the entire 36,379-molecule library to the MAP38 kinase. (B) Enrichment curve for the top 100 docked compounds to MAP38 kinase. (C) Enrichment curve for ModBind, using the top 100 docked compounds to model dissociation of small molecules from MAP38 kinase. (D) Enrichment curve for docking of the entire 11,398-molecule library to the MET kinase. (E) Enrichment curve for the top 100 docked compounds to MET kinase. (F) Enrichment curve for ModBind, using the top 100 docked compounds to model dissociation of small molecules from MET kinase. ROC: receiver operator characteristics; enrichment factor 5%: enrichment of actives over decoys using 5% of the decoy library, as given by EF = (*a*/*n*)/(*A*/*N*), where *a* is the number of actives found in sample size *n*, *A* is the total number of actives, and *N* is the total number of ligands (decoys and actives).

Here, we tested ModBind’s ability to increase enrichment of true positive hits using compound lists that were preprioritized via traditional VS. We took the DUD-E data set^48^ and selected the MAP38 kinase compounds which were docked to the MAP38 protein using AutoDock Vina.^31,49^ To mimic a typical VS campaign that would use docking results to inform compound selection for subsequent synthesis and testing, we took the top 100 scoring compounds (out of a total of 36,379 docked compounds), consisting of 20 active compounds and 80 decoy compounds, re-evaluated them in ModBind, and compared the resulting enrichment to the docking results. If we use docking scores from the entire 36,379-compound data set, enrichment of active compounds over decoy compounds follows an expected trend, with an enrichment factor of 5.8 (Figure 4A). However, when we look at the top 100 docked results, differentiation between actives and decoys is negligible, leading to an enrichment factor of 2.1 (Figure 4B). In contrast, ModBind vastly outperforms docking, showing a 27-fold enrichment within the top 100 compounds by correctly identifying nearly 80% of the actives before ranking any decoys (Figure 4C). We performed the same analysis on the MET kinase data set and saw a similar trend: docking-improved enrichment of the entire compound library (Figure 4D), poor enrichment of the top 100 compounds (2.9) (Figure 4E), and significantly improved enrichment with ModBind (20) (Figure 4F). In numerous in-house studies on other protein targets, we find that reranking top docked compounds with ModBind consistently and significantly outperforms docking alone. Thus, ModBind would be an invaluable tool to postprocess VS results and increase both hit rate and efficiency of the hit-to-lead workflow.

### ModBind in Practice

ModBind has been used on Alivexis’ internal projects since its inception. Multiple proprietary series of compounds have been developed, with several series in late-stage lead optimization for the internal project discussed here. Through the later stages of this internal campaign, we have used ModBind to rapidly and efficiently narrow down selection of compounds for synthesis. For validation in this particular project, thirty-six compounds were selected retrospectively for runs in ModBind with a broad array of activity and modifications in the R-groups. The results of the validation run showed a good correlation between experimental and predicted values. We proceeded to use ModBind to guide the prospective predictions of hundreds of possible compounds. To date, 45 compounds have been synthesized from the prospectively predicted compounds through ModBind.

**Figure 5:**
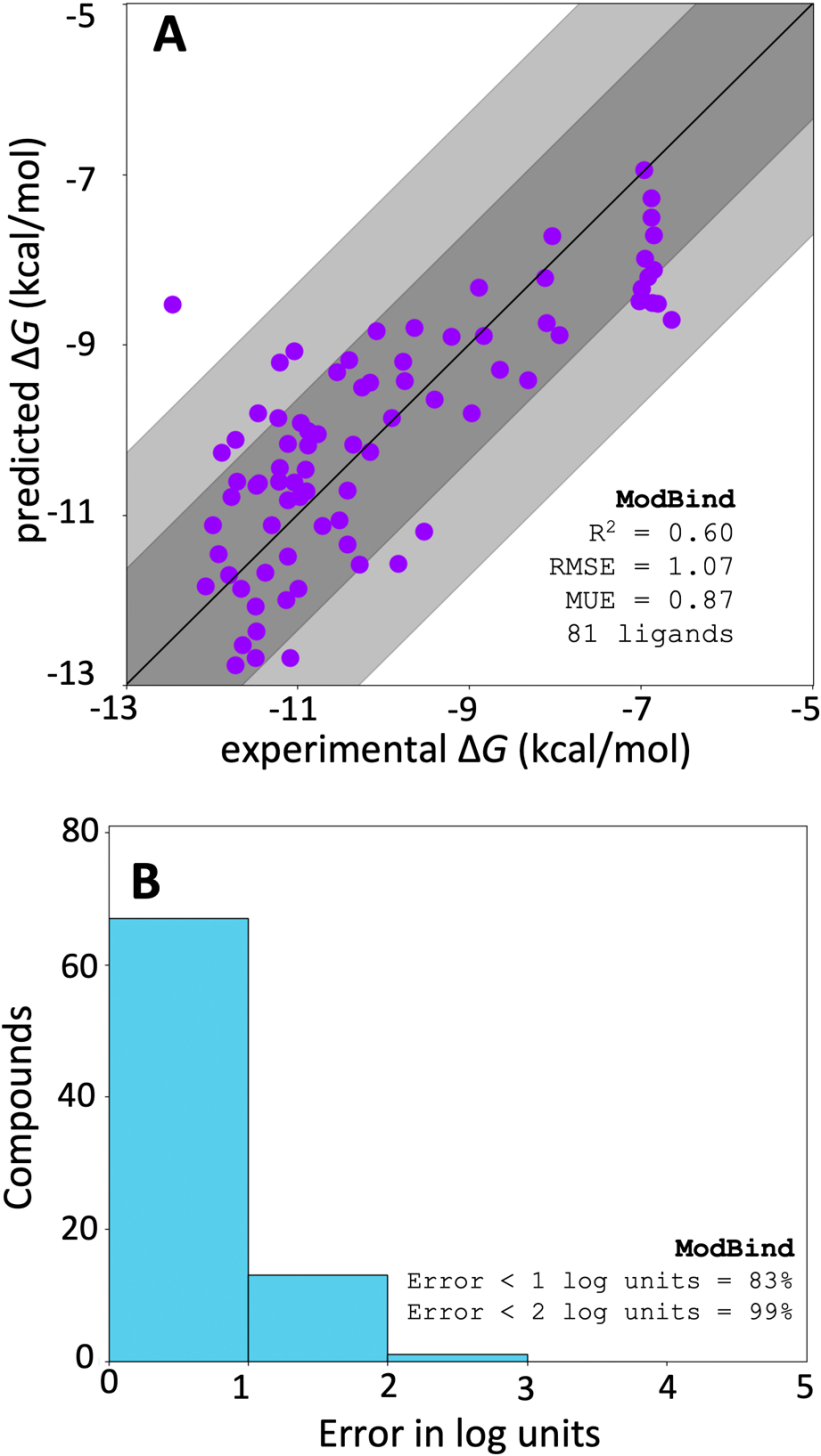
ModBind prospective and retrospective predictions for an internal project. (A) ModBind predictions compared with experimental Δ*G* of binding for retrospective and prospective inhibitors. Dark-gray shading indicates less than 1 log order error, and light-gray shading indicates less than 2 log order error. (B) Bar chart of errors between ModBind and experiment for retrospective and prospective predictions. RMSE: root-mean-squared error; MUE: mean unsigned error.

ModBind predicted binding energies with comparable accuracy to that of our previous validation work on other targets, showing good correlation with experimental results and low levels of error (Figure 5A). Note that these compounds were chosen prospectively via ModBind and thus we observed a systematic overprediction bias;^50^ the predicted errors are low, with nearly 83% of predictions within 1 log unit and 99% of predictions within 2 log units (Figure 5B). Seventy six percent of prospective predictions less than 10 nM (less than −10.9 kcal/mol) were confirmed to have corresponding experimental affinities (<10 nM) in our biochemical assay. This level of accuracy is in the range of the best free-energy methods^51^ and sufficient to guide our medicinal chemistry synthesis decisions. In comparison to alchemical free-energy predictions and other related simulation-based methods, the computational turnaround time for ModBind calculations was approximately 100 times faster (Table 2).

**Table 2:** Approximate Timings of Relative^3,5,52^ and Absolute^44,53,54^ Alchemical Free Energy and ModBind Predictions Based on the Cited Literature^a^

Methodology	nanoseconds/simulation/compound	estimated compounds/day/GPU	suitable for VS and screening diverse ligands
relative alchemical free energy	240–480	2–4	No
absolute alchemical free energy	640–1240	1–2	Yes
ModBind virtual screen	4	250	Yes

a Estimated compounds/day/GPU is based on a GPU that achieves a currently reasonable speed of 1000 ns/day.

In addition to using ModBind in our later stage programs, we have prospectively tested ModBind as a filter in the later steps in the large-scale VS using a random chemical library. We have run multiple docking-based virtual screens prior to the development of ModBind. These relied on the same general workflow of docking millions of purchasable compounds and then purchasing a few hundred compounds for in vitro testing. These virtual screens had success, in some cases identifying highly valuable leads.^55,56^ For our docking-based virtual screens, we discovered novel cores with a measurable experimental affinity less than 100 μM with an average hit rate of 1.3 novel cores per virtual screen (Table 3). Part of our motivation to develop ModBind was to allow us to have much greater hit rates of novel cores in VS. When we used ModBind at the end of a virtual screen to rank the top ∼20,000 compounds, selected based on the docking score and structural diversity, we saw a significant increase in the hit rate. Our docking plus ModBind virtual screen saw the identification of 14.0 novel cores with IC_50_ values less than 100 μM per virtual screen as an average.

**Table 3:** Summary of the Number of Novel Compound Cores Found on Average for Virtual Screens Performed at Alivexis, Inc

methodology	average novel compound cores identified <100 μM per VS
docking	1.3
docking + ModBind	14.0

**Figure 6:**
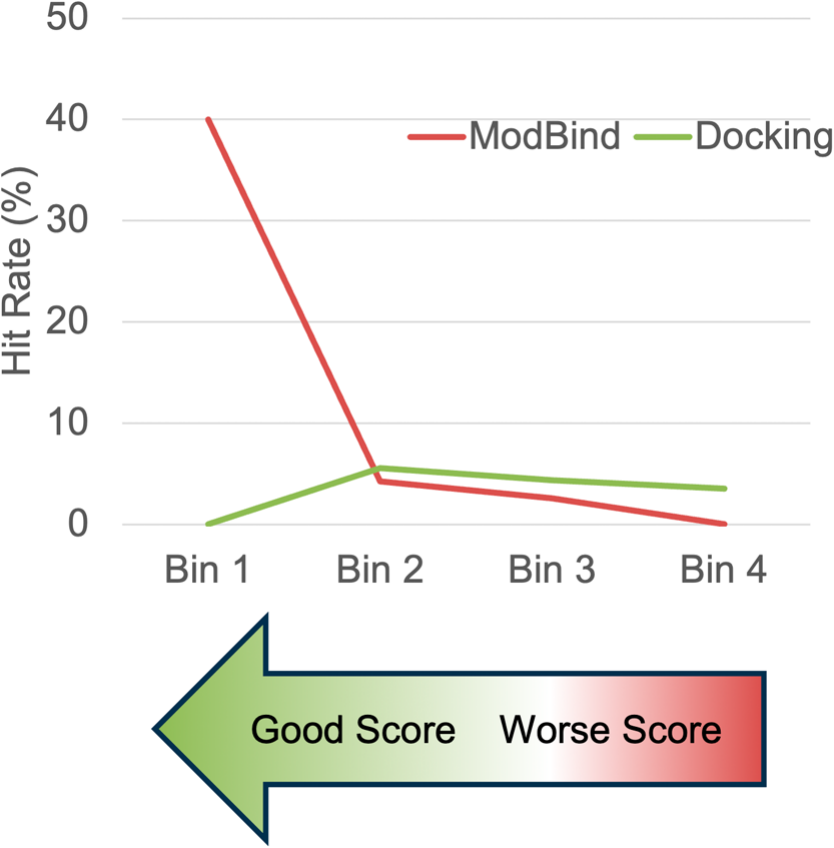
Hit extraction rates based on binning of docking scores and ModBind scores for our docking + ModBind virtual screen.

Some SAR trends could be seen among series of hit compounds and many weaker inhibitors were found as well. When we reviewed our VS hits, we also noted a trend indicating that better ModBind scores led to higher hit rates, up to 40%, and lower ModBind scores led to lower overall hit rates. Although docking was critical for posing the compounds prior to a ModBind prediction, better docking scores showed no increase in the hit rate (Figure 6). Although docking is useful for the general enrichment of virtual libraries, a more accurate computational tool such as ModBind applied to the top docking hits significantly improves the outcome of virtual screens. The VS workflow that included docking followed by ModBind ranking produced a diverse array of starting chemical leads, which is an excellent starting point for any drug discovery program. Our virtual screens and lead optimization programs utilized assays based on luminescence, fluorometric, fluorescence resonance energy transfer, radiometric, fluorescence polarization, and colorimetric methodology. We have not seen a significant difference in ModBind accuracy based on experimental assay methodology in our data, indicating the broad applicability of our predictions.

In general, ModBind is much faster to run (10–100 times or greater) than alchemical free-energy methods. In this work, we have run ModBind with varying settings including the temperature, length of simulation, and number of replicas (between 8 and 32). For example, the HSP90 work used on average 13 ns of total simulation per ligand, and our docking + ModBind workflow (Table 3) used on average 4 ns of total simulation per ligand for >20,000 ligands. Given that published relative alchemical methods require between a few hundred nanoseconds of total simulation time^3,5,52^ and absolute alchemical methods require up to 1000 ns of total simulation time^44,53,54^ per ligand, we can achieve up to 2 orders of magnitude greater ligand predictions with the same GPU resources. Given that the fastest current GPUs are providing thousands of nanoseconds of simulation per day (based on the DHFR benchmark^57^), we can predict hundreds of compounds per day or more on fast GPUs (Table 2). ModBind is trivially parallelizable, allowing us to easily scale up and predict tens of thousands of compounds or more in relatively short time, and we reasonably expect that as hardware and software improve the speed and accuracy of MD methods, ModBind’s speed and accuracy will also improve. Previously, we have proposed that the calculation of free energies converges more rapidly for population-based methods,^23^ and we believe that ModBind as a population-based method combined with an optimized workflow simultaneously realizes significantly higher speed and greater accuracy.

## Conclusions

Here, we have presented ModBind, a new enhanced sampling method for the prediction of ligand off-rates. The methodology is firmly rooted in statistical mechanics and has been validated across more than ten targets and hundreds of ligands. We also showed how ModBind was successfully used prospectively to screen tens of thousands of ligands in our internal drug discovery campaigns at Alivexis. ModBind accelerates ligand dissociation and can accurately capture unbinding kinetics within half a log unit, all with a cumulative simulation time of as little as 4 ns per ligand. In addition, we showed that for a large variety of protein targets and compound sets, ModBind can be used to indirectly predict binding affinities or IC_50_ values. ModBind is at least 10–100 times faster than the majority of free-energy implementations but maintains the same degree of accuracy. A major advantage of ModBind is that it does not use alchemical changes to obtain results, negating the requirement for a reference compound that is inherent in the relative FEP approaches. This allows for de novo design of compounds and absolute comparisons between diverse ligands in a VS application. Although *k*_off_ rates are not always directly correlated with *K*_d_ (and thus free energies), in many cases, ModBind can be used to identify potent and efficacious compounds, with the added potential benefit of an increased *k*_off_ value, which is often a desirable pharmacological property in its own right. We demonstrated the utility of ModBind calculations in VS and lead optimization of our internal programs. With the ease of implementation using almost any MD engine, the ease of access to GPU resources, and the low computational requirement of the method, we envision ModBind becoming an important tool in almost any computational drug discovery program moving forward. Further improvements to ModBind, including the direct prediction of absolute binding free energies and selection of binding poses, are currently forthcoming.

## Data Availability

Machine-readable files for the 3D chemical structures and protein structures needed to perform our entire validation can be found in the Supporting Information (ZIP).

## Supporting Information Available

Sources of all chemical structures and protein structures used to run the validation simulations and a description of the data normalization procedure (PDF)
3D chemical structures and protein structures used in ModBind validation (ZIP)

## Abbreviations

MD: molecular dynamics
rmsd: root-mean-squared deviation
*NVT*: constant number atom volume and temperature
MAPK: mitogen-activated protein kinase
FAK: focal adhesion kinase
HSP90: heat shock protein 90
MUE: mean unsigned error
RMSE: root-mean-squared error
FEP: free-energy perturbation
TI: thermodynamic integration
TYK2: tyrosine kinase 2
MCL1: myeloid cell leukemia 1
JNK1: c-Jun N-terminal kinase 1
CDK2: cyclin-dependent kinase 2
PTP1B: protein tyrosine phosphatase 1B
BACE: β-site amyloid precursor protein-cleaving enzyme
VS: virtual screening
