# Supplementary material for: ModBind, a Rapid Simulation-Based Predictor of Ligand Binding and Off-Rates
Source: J Chem Inf Model. 2024 Dec 16;65(1):265–74. doi: 10.1021/acs.jcim.4c01805 (PMC11733936; doi:10.1021/acs.jcim.4c01805)
Supplement: Supplementary file 1 — ci4c01805_si_001.pdf [file ci4c01805_si_001.pdf]

Supplemental Information

ModBind™, a rapid simulation-based predictor of ligand off-rates

William Sinko[1*], Blake Mertz[1], Takafumi Shimizu[2], Taisuke Takahashi[2], Yoh Terada[2], S. Roy Kimura[2]

1. Alivexis Inc. 1 Broadway, 14th Floor, Cambridge, MA 02142
2. Alivexis Inc. Daiichi Hibiya Building 7F, Shimbashi 1-18-21, Minato-ku, Tokyo 105-0004

* Email: sinko@alivexis.com

Sources for structures of ligands and proteins used in this study:

Off-rate predictions. For P38 MAPK the data was obtained from *Regan et al.*[2] with structures curated from *Georgi et al.*[3] For HSP90, the ligands and protein structures were taken from *Ganotra et al.* [1] The ligand and protein structures can be found in the supplemental information of the original publication by *Ganotra et al*[1] and the ligand structures are shown here. We used the structure PDB 1KV2 as the protein structure for the ligand bound simulations[4]. For FAK we used chemical structure and protein structures PDB 4GU6 data from Heinrich et al.[5]

Free energy predictions. All chemical structure data and protein structures for CDK2, Thrombin, P38 MAPK, JNK1, TYK2, and MCL1 was obtained from Wang et al[6] This data is freely available on github https://github.com/schrodinger/public_binding_free_energy_benchmark. This data is curated in 3D and ready for use in molecular simulations.

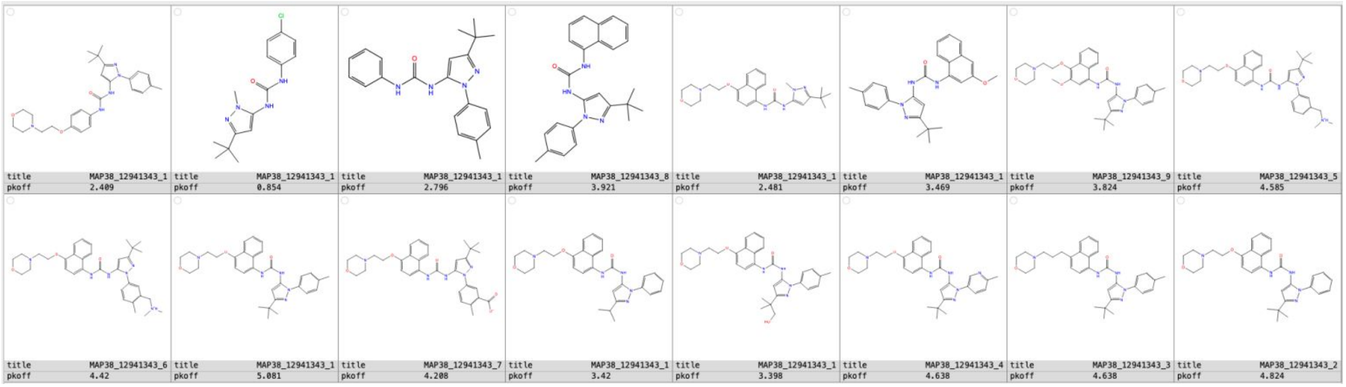

Supporting Figure S1. P38 MAPK ligands from Regan *et al.* and pK_{off} rate

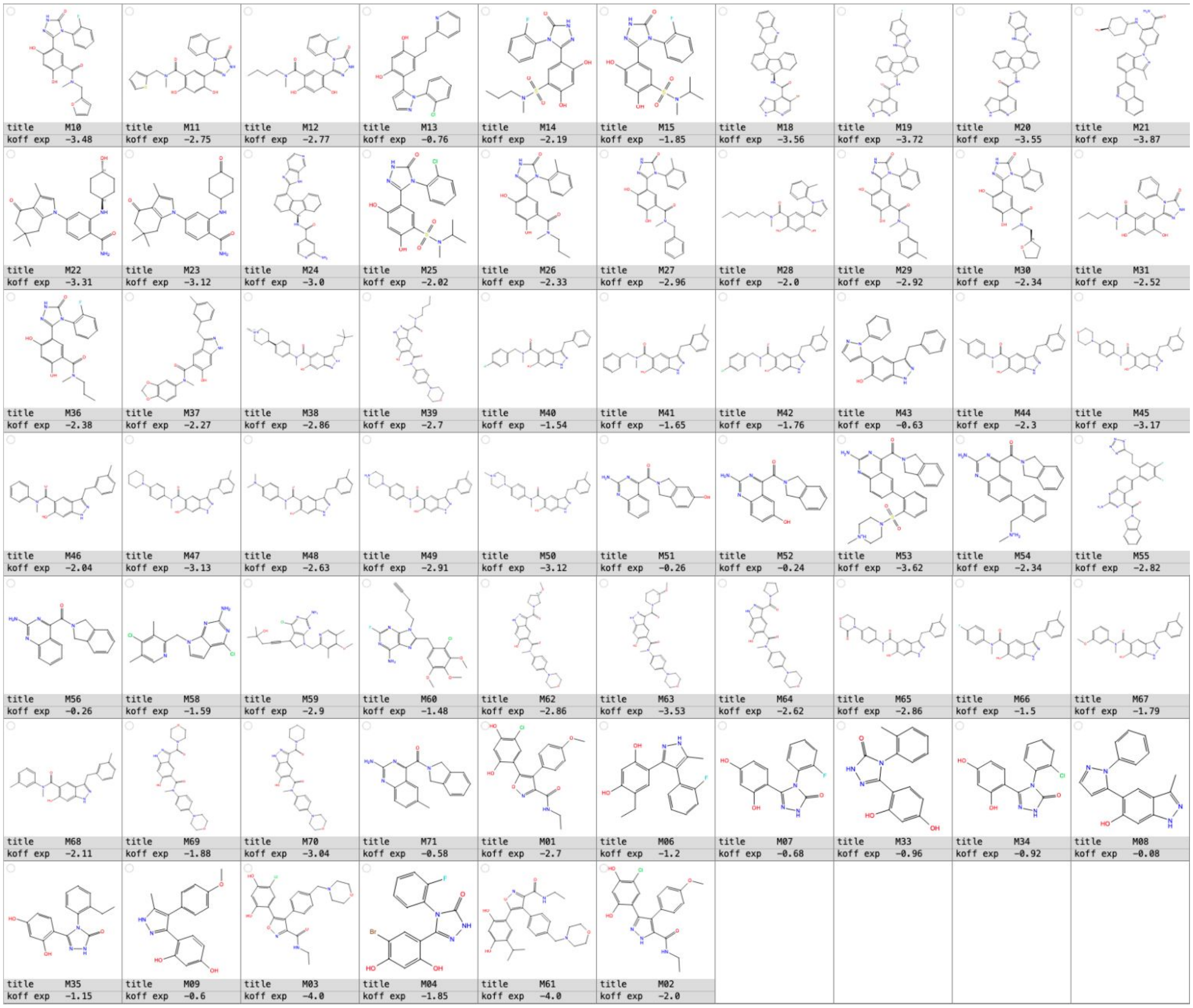

Supporting Figure S2. HSP90 Ligands from Ganotra *et al.* and pK_{off} rate.

.

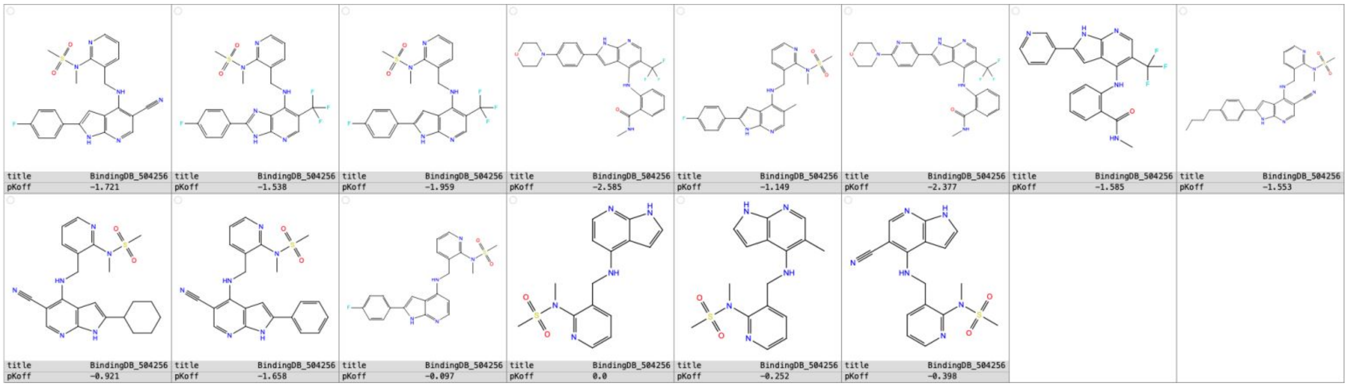

Supporting Figure S3. FAK ligands from Heinrich *et al.* and pK_{off} rate.

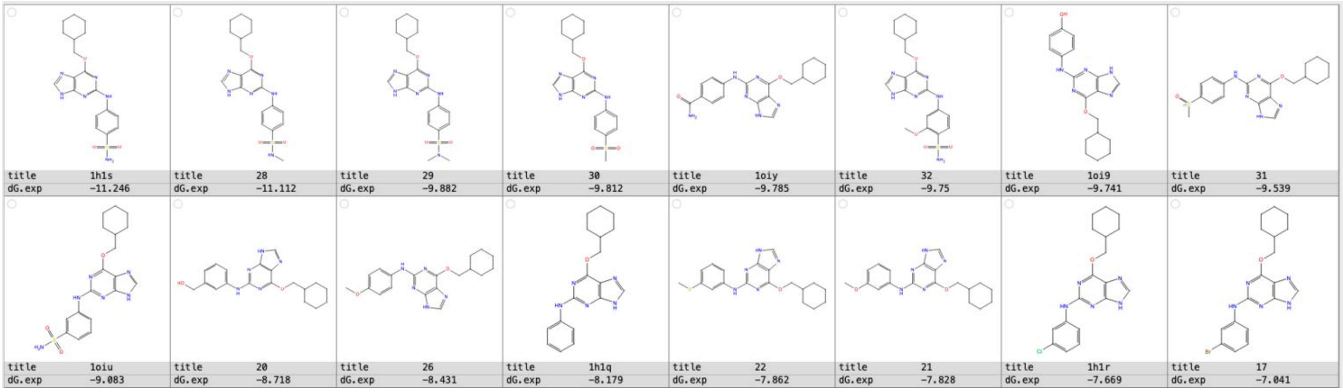

Supporting Figure S4. CDK2 ligands from Wang *et al.* and Δ*G* of binding.

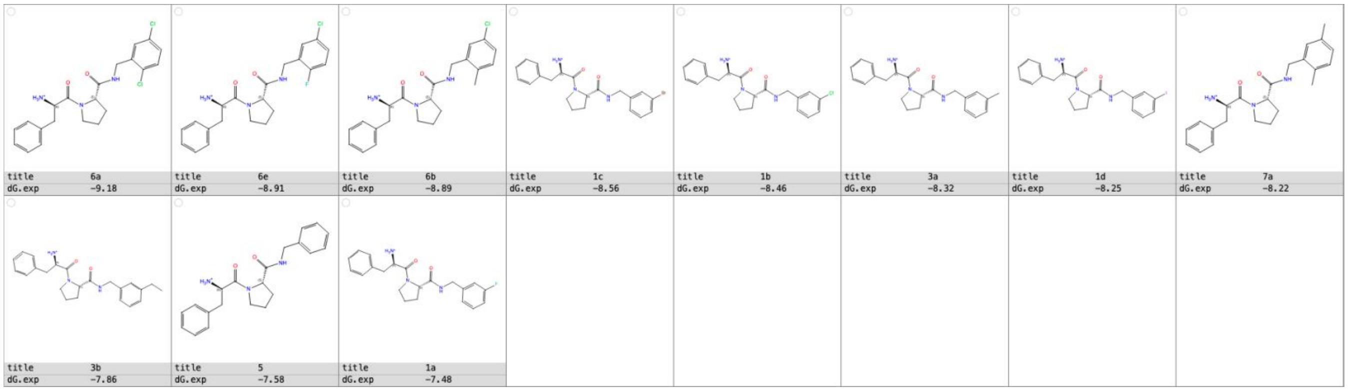

Supporting Figure S5. Thrombin ligands from Wang *et al.* and Δ*G* of binding.

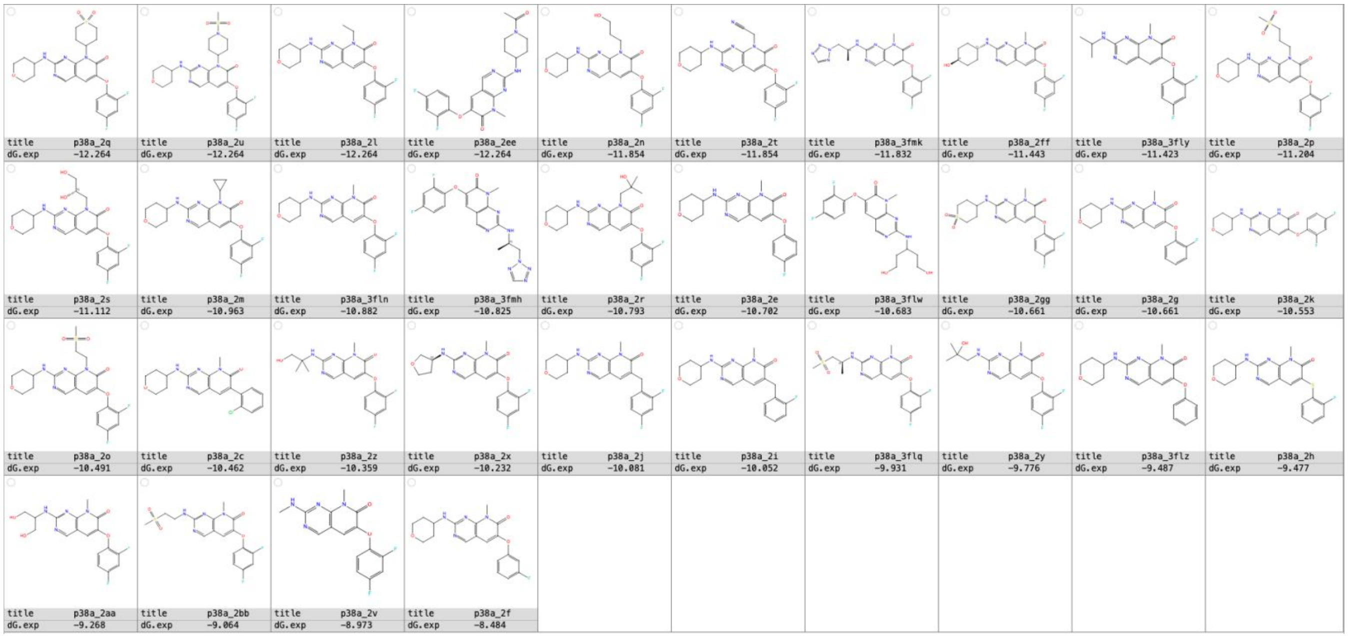

Supporting Figure S6. P38 MAKP ligands from Wang *et al.* and Δ*G* of binding.

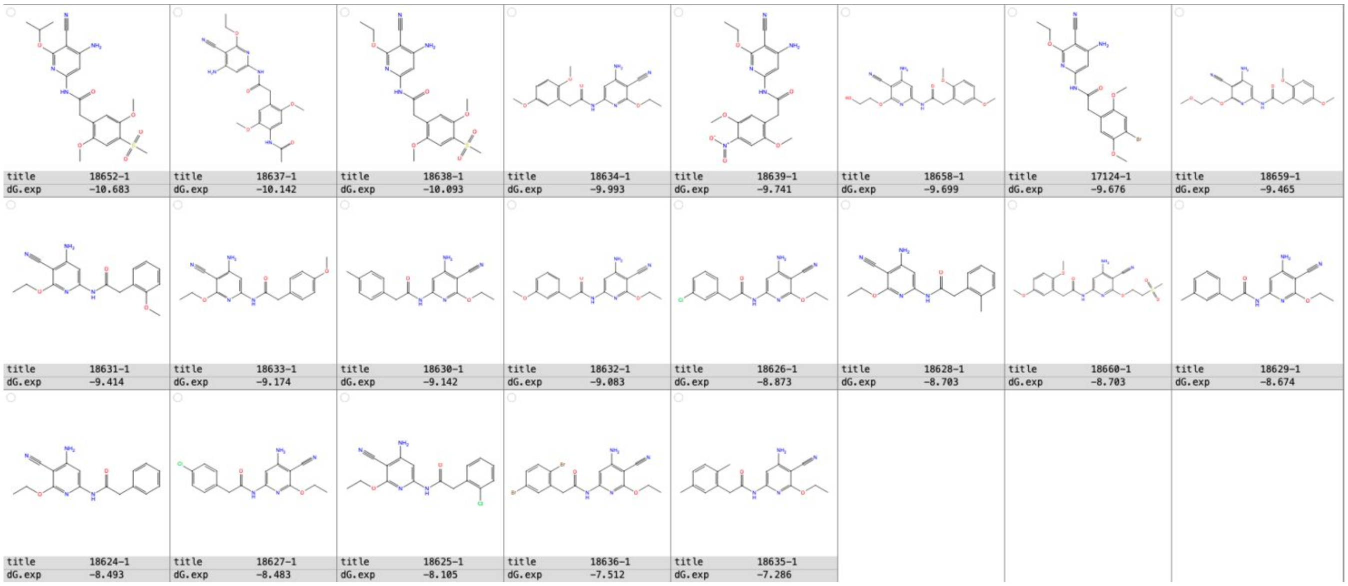

Supporting Figure S7. JNK1 ligands from Wang *et. al*. and Δ*G* of binding.

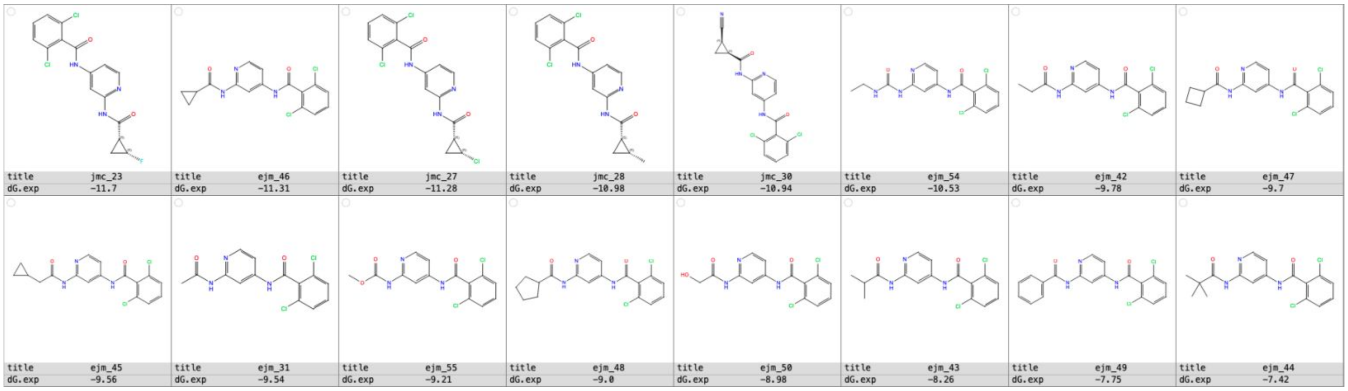

Supporting Figure S8. TYK2 ligands from Wang *et al.* and Δ*G* of binding.

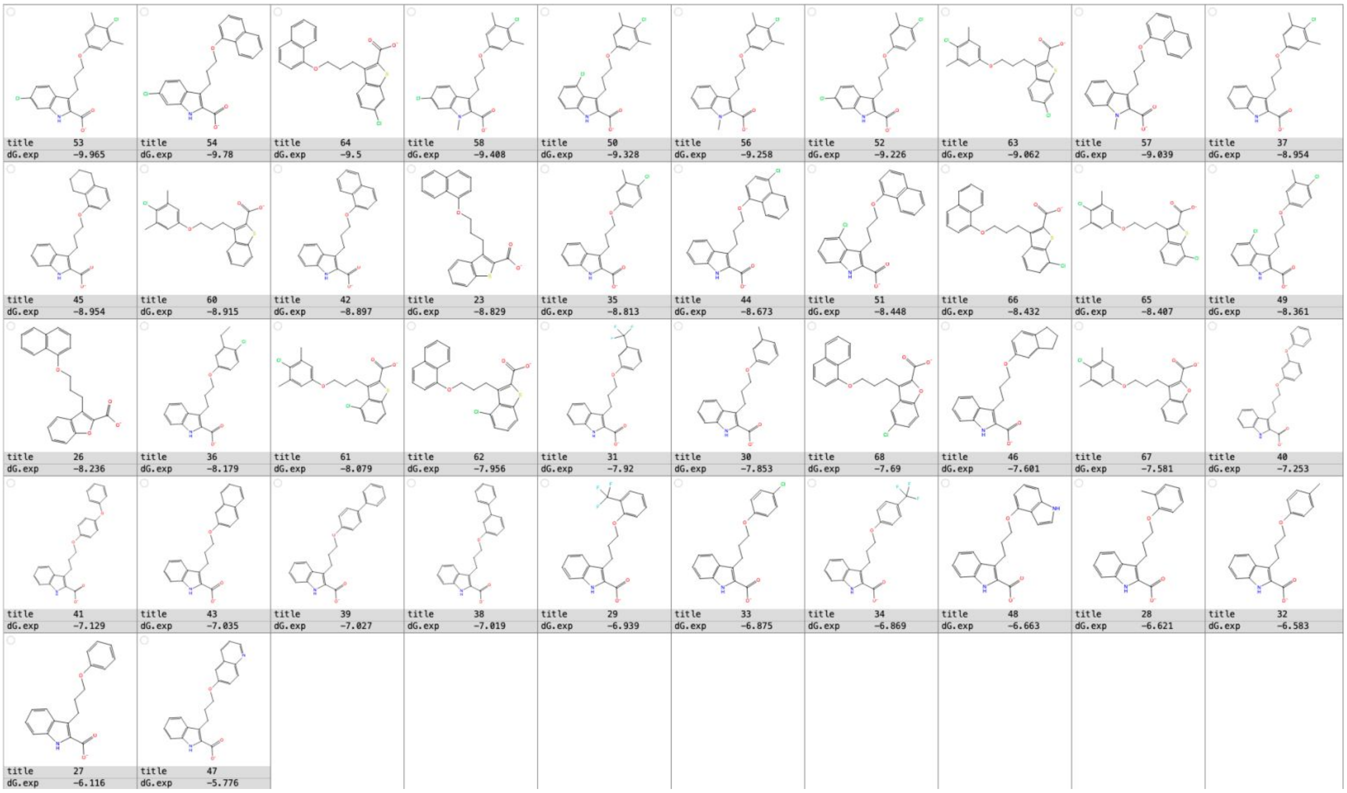

Supporting Figure S9. MCL1 ligands from Wang *et al.* and Δ*G* of binding.

Normalization procedure for graphing predicted data and experimental data. We have normalized the data generated from ModBind or other computational methods. This is required to give reasonable error estimates since the ModBind predictions are on another scale as compared to the experimental methods especially related to free energy. We first normalize the means of the data setting the two to be equivalent. Then we normalize the range of the data to be equivalent. Relative binding calculations like FEP or AMBER TI normalize the mean of the data for any dataset, since they only give relative changes between ligands, rankings for each technique stays consistent across all methods. In all cases in this paper we have applied the same normalization procedure to both ModBind and other known methods like FEP, AMBER-TI, and docking in order to have consistent and accurate comparison of errors. Correlation metrics are unchanged by the procedure.

(1) Ganotra, G. K.; Wade, R. C. Prediction of Drug–Target Binding Kinetics by Comparative Binding Energy Analysis. *ACS Med. Chem. Lett.* **2018**, *9* (11), 1134–1139. https://doi.org/10.1021/acsmedchemlett.8b00397.

(2) Regan, J.; Pargellis, C. A.; Cirillo, P. F.; Gilmore, T.; Hickey, E. R.; Peet, G. W.; Proto, A.; Swinamer, A.; Moss, N. The Kinetics of Binding to p38MAP Kinase by Analogues of BIRB 796. *Bioorg. Med. Chem. Lett.* **2003**, *13* (18), 3101–3104. https://doi.org/10.1016/s0960-894x(03)00656-5.

(3) Georgi, V.; Schiele, F.; Berger, B.-T.; Steffen, A.; Marin Zapata, P. A.; Briem, H.; Menz, S.; Preusse, C.; Vasta, J. D.; Robers, M. B.; Brands, M.; Knapp, S.; Fernández-Montalván, A. Binding Kinetics Survey of the Drugged Kinome. *J. Am. Chem. Soc.* **2018**, *140* (46), 15774–15782. https://doi.org/10.1021/jacs.8b08048.

(4) Pargellis, C.; Tong, L.; Churchill, L.; Cirillo, P. F.; Gilmore, T.; Graham, A. G.; Grob, P. M.; Hickey, E. R.; Moss, N.; Pav, S.; Regan, J. Inhibition of P38 MAP Kinase by Utilizing a Novel Allosteric Binding Site. *Nat. Struct. Biol.* **2002**, *9* (4), 268–272. https://doi.org/10.1038/nsb770.

(5) Heinrich, T.; Seenisamy, J.; Emmanuvel, L.; Kulkarni, S. S.; Bomke, J.; Rohdich, F.; Greiner, H.; Esdar, C.; Krier, M.; Grädler, U.; Musil, D. Fragment-Based Discovery of New Highly Substituted 1H-Pyrrolo[2,3-b]- and 3H-Imidazolo[4,5-b]-Pyridines as Focal Adhesion Kinase Inhibitors. *J. Med. Chem.* **2013**, *56* (3), 1160–1170. https://doi.org/10.1021/jm3016014.

(6) Wang, L.; Wu, Y.; Deng, Y.; Kim, B.; Pierce, L.; Krilov, G.; Lupyan, D.; Robinson, S.; Dahlgren, M. K.; Greenwood, J.; Romero, D. L.; Masse, C.; Knight, J. L.; Steinbrecher, T.; Beuming, T.; Damm, W.; Harder, E.; Sherman, W.; Brewer, M.; Wester, R.; Murcko, M.; Frye, L.; Farid, R.; Lin, T.; Mobley, D. L.; Jorgensen, W. L.; Berne, B. J.; Friesner, R. A.; Abel, R. Accurate and Reliable Prediction of Relative Ligand Binding Potency in Prospective Drug Discovery by Way of a Modern Free-Energy Calculation Protocol and Force Field. *J. Am. Chem. Soc.* **2015**, *137* (7), 2695–2703. https://doi.org/10.1021/ja512751q.
